# Trimodality therapy of esophagectomy plus neoadjuvant chemoradiotherapy improves the survival of clinical stage II/III esophageal squamous cell carcinoma patients

**DOI:** 10.3892/or.2012.1847

**Published:** 2012-06-01

**Authors:** YOSHINORI FUJIWARA, REIGETSU YOSHIKAWA, NORIHIKO KAMIKONYA, TSUYOSHI NAKAYAMA, KOTARO KITANI, MASANORI TSUJIE, MASAO YUKAWA, MASATOSHI INOUE, TAKEHIRA YAMAMURA

**Affiliations:** 1 Department of Digestive Surgery, Nara Hospital, Kinki University School of Medicine, Nara; 2 Department of Surgery, Kanzaki Hospital, Hyogo; 3 Department of Radiology, Hyogo College of Medicine, Nishinomiya, Japan; 4 Department of Surgery, Hyogo College of Medicine, Nishinomiya, Japan

**Keywords:** chemoradiotherapy, esophagectomy, survival

## Abstract

The prognosis of advanced esophageal cancer patients is poor. Trimodality therapy of surgical resection plus neoadjuvant chemoradiotherapy (CRT) has been developed to improve survival through locoregional control, leading to prevention of micrometastasis. We investigated whether or not neoadjuvant CRT led to survival benefits in TNM stage II/III esophageal cancer patients. We retrospectively reviewed 62 patients with stage II or III esophageal squamous cell carcinoma (ESCC) treated with neoadjuvant CRT. All patients received esophagectomy 4–7 weeks after CRT consisting of 40 Gy irradiation and chemotherapy (5-FU, 500 mg/m^2^/day, days 1–5 and cisplatin, 10–20 mg/body, days 1–5). Clinical response and survival rates were analyzed using Kaplan-Meier methods, with P<0.05 considered as significant. The clinical effect rate of CRT for both primary tumors and metastatic nodes was 82.3%. Operative and hospital mortality rates were 1.65 and 6.5%, respectively. The 3-year overall survival (OS) and disease-free survival (DFS) rates were 52.6 and 49.2%, respectively. A significant difference was noted between stages II and III for both OS and DFS. The 5-year OS rates were 64.2% for stage II, 33.1% for stage III (T4 and non-T4) and 46.9% for stage III (non-T4 only) patients. The depth of tumor invasion (T3 vs. T4), resectability (R0 vs. R1, R2), lymph node metastasis (positive vs. negative), and the effect of CRT were proven to be independent prognostic factors for univariate analysis, with resectability and the effect of CRT for multivariate analysis. These data suggest that CRT in stage II/III (non-T4) ESCC patient contributed to tumor shrinkage, leading to higher resectability and longer survival. Neoadjuvant CRT appears to be a promising option for these patients.

## Introduction

Esophageal cancer is one of the most aggressive malignancies and is associated with a poor prognosis because of early metastasis to lymph nodes as well as distant organs (1–3). Esophageal squamous cell carcinomas (ESCCs) are far more common in Asian countries including Japan, whilst adenocarcinomas of the lower third of the esophagus are often seen in Western countries. In 2005, 11,182 Japanese died from esophageal cancer according to the Japanese Ministry of Health, Labour and Welfare. Surgery has been considered the treatment of choice for patients with locoregionally confined esophageal carcinoma. However, the 5-year survival rate is less than 25% worldwide (4–6).

In Japan, the survival rate has been improving during the past two decades since three field lymphadenectomy was advocated by Isono *et al*(7) and Akiyama *et al*(8) and it is now widely performed. According to the comprehensive registry of esophageal cancer in Japan (3rd edition) (9), the current survival rates of clinical stage IIA, IIB and III patients categorized by UICC (10) are reportedly 47.5, 45.1 and 33.3%, respectively. These results were rather disappointing in spite of vigorous lymphadenectomy. The bottom-line in esophageal cancer treatment is locoregional control, and the locoregional failure rate after esophagectomy has been reported to be approximately 30% for patients who received R0 resection (11). Likewise, the locoregional recurrence rate (which included persistent disease and locoregional recurrence) is 50–55% after definitive chemoradiotherapy (CRT) without surgery (12,13).

To resolve these locoregional failures, multimodality therapy involving the combination of surgery and CRT has been developed. The most common approach is preoperative CRT followed by esophagectomy, called trimodality therapy (14,15). This approach offers the potential advantage of tumor downstaging, less dissemination of malignant cells during surgery and prevention of micrometastasis. Nine randomized trials have been performed in patients with locoregionally confirmed esophageal cancer who received preoperative CRT compared with surgery alone (15–23). Two of these 9 studies showed an improved outcome despite a small number of patients (15), while the other studies showed no survival benefits in the trimodality therapy group. Therefore, the benefits of preoperative CRT are still controversial.

There is no randomized study ongoing or being planned related to preoperative CRT of ESCC in Japan because of technical difficulties both in surgery and radiotherapy. Since 1996, we have introduced preoperative CRT using 5-fluorouracil (5-FU) and cisplatin (CDDP) combined with radical surgery for the treatment of advanced esophageal cancers, and have reported increased resectability, a reduced incidence of both local recurrence and distant metastasis, and a more favorable prognosis for CRT responders (24). In the present study, we re-evaluated the feasibility and efficacy of preoperative CRT and investigated whether a survival benefit was obtained for stage II/III ESCC patients receiving neoadjuvant CRT.

## Patients and methods

### Patients

We performed a retrospective review of 80 consecutive patients with esophageal cancer who received esophagectomy after neoadjuvant CRT between August 1997 and October 2007 at the Department of Surgery, Hyogo College of Medicine, Japan. Sixty-two of the 80 patients had clinical stage II or III disease based on the UICC TNM Classification of Malignant Tumors (5th edition) (10), as determined by CT scan and/or endoscopic ultrasound examination findings, and underwent concurrent CRT followed by esophagectomy.

The eligibility criteria of this study were as follows: <80 years old, adequate organ function (WBC≥3500, Hb≥10 g/dl, ALT/AST≤2× upper limit of normal, platelets ≥100,000, serum creatinine≤1.3), and a performance status (Eastern Cooperative Oncology Group) of <2 at the time of admission (Table I).

Preoperative radiotherapy was performed for 5 days per week (Monday to Friday, 2 Gy/day) using a linear accelerator (Mevatron KD2; Siemens, Germany). The radiation field encompassed the primary tumor volume (as defined by endoscopy, esophagography and CT scan) with a 3-cm margin in each cephalad and caudal direction and 4-cm horizontal margins. If the lymph nodes metastasis was detected by a CT scan, the radiation field was extended to include the primary tumor and metastatic lesions. The patients received 20 fractions of 2 Gy for a total of 40 Gy of radiation. Concurrent chemotherapy consisted of 5-FU (500 mg/m^2^/day) administration for a 120-h continuous intravenous infusion starting on Day 1 and CDDP (15–20 mg/day) for a 2-h intravenous infusion on Days 1–5, repeated after 3 weeks.

Two to three weeks after the completion of radiotherapy, the effects of CRT on the primary tumor and metastatic nodes were assessed using chest CT scanning, barium esophagography, and/or upper gastrointestinal endoscopy. The response to therapy was defined as confirmed by esophagography or esophagoscopy and CT scans according to the criteria of the Japanese Society of Esophageal Disease (9th edition) (25): i) complete response (CR), 100% regression of cancer; ii) partial response (PR), >50% regression of the primary tumor and metastatic nodes; iii) progressive disease (PD), defined as increase of 25% in the size of the primary tumor or metastatic nodes or the appearance of new lesions; and iv) no change (NC) defined as a decrease of <50% in the size of the primary tumor and metastatic nodes and no evidence of tumor progression. Toxicities were classified according to NCI CTC Guidelines, version 3 (26).

Esophagectomy was planned for 4–7 weeks after the completion of CRT. Most patients underwent thoracotomy, laparotomy, and cervicotomy to perform esophagectomy with 2- or 3-fields lymphadenectomy, and gastroesophageal anastomosis at the left side of the neck. Radical resection (R0) was defined as the removal of all macroscopic tumors, no evidence of distant metastasis, the absence of a microscopic residual tumor, free resection margins, and lymphadenectomy extending beyond the involved nodes. Resection was defined as non-radical when a microscopic (R1) or macroscopic (R2) residual tumor was found according to the TNM criteria (10). Informed consents were obtained in all patients.

### Statistical analysis

Overall survival (OS) was defined as the time from the data of initial treatment to patient death or the data of the last available information on the vital status. Disease-free survival (DFS) was defined as the length of time after treatment during which no cancer was found. Differences between the cumulative survival rates of the patient groups were calculated by the log-rank test for comparison using Kaplan-Meier survival curves. Statistical significance was considered at values of P<0.05. Univariate analyses were used to examine the patients’ characteristics and other prognostic factors. Multivariate analyses were employed for the identification of prognostic factors with the Cox proportional hazard model. Statistical analyses were carried out using the Statistica software, version 06J (StatSoft, Tulsa, OK, USA), and SPSS version 16 (SPSS, Tokyo, Japan).

## Results

### Patient characteristics

The patient characteristics of this study are summarized in Table I. All tumors were histologically confirmed to be ESCC. The gender was biased toward males (male/female, 50:12). The mean age was 60.83 years old. Fifty-eight of the 62 patients had tumors in the thorax. Seventeen stage III patients had tumors infiltrating through the esophageal wall to adjacent structures (T4, 53.1% of stage III patients). Nineteen patients (30.65%) had lymph nodes metastasis on a CT scan at the time of diagnosis.

### Response and toxicities

The clinical response to CRT is summarized in Table II. The clinical response (CR+PR) rates of CRT for the primary tumor and metastatic nodes were 83.9 and 70%, respectively. The clinical response of both the primary tumor and metastatic nodes was 82.3%. Major toxicities of treatment are summarized and laboratory findings were obtained from 59 patients. Leukocytopenia and thrombocytopenia of grade 3 or higher were noted in 33.9 and 5.1% of the patients, respectively. Liver dysfunction of grades 1 or 2 was noted in 11.8%. Fatigue, stomatitis and nausea of grade 1 or 2 were noted in 36, 10 and 26% of the cases, respectively. Other toxicities were found in 50 patients. CRT-related death was not reported.

### Surgery and postoperative complications

All patients underwent esophagectomy after the completion of CRT. Radical R0 resection was achieved in 45 patients (72.6%), R1 resection with a microscopic residual tumor was achieved in 8 (12.9%), and R2 resection with a macroscopic residual tumor in 9 (14.5%). The reasons for the failure of radical resection leading to R2 resection were a residual primary tumor in 4 patients, metastatic nodes in 3, and the occurrence of new distant metastasis during neoadjuvant CRT in 2. Postoperative complications are shown in Table III. One patient died from occlusion of the superior mesenteric artery (SMA) within 4 weeks of surgery, corresponding to 1.6% of the operative mortality. Three patients died of respiratory failure including 2 metastatic lung cancers within 3 months after the operation corresponding to 6.5% of hospital mortality.

### Pathological response of the primary tumor

Fifteen of the 62 patients (24.2%) had no residual tumor in the resected esophagus, representing pathological CR.

### Survival

**Figure 1:**
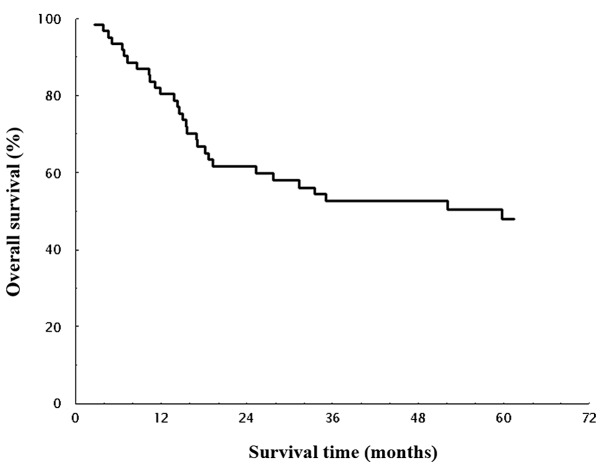
Overall survival in stage II and III esophageal cancer patients.

**Figure 2:**
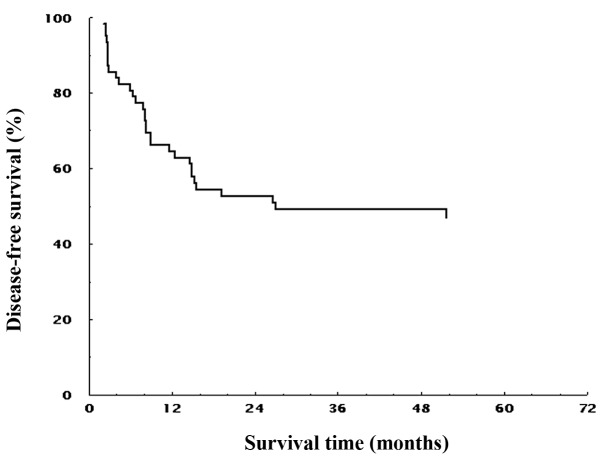
Disease-free survival in stage II and III esophageal cancer patients.

The mean follow-up period was 46 months (3–169 months). OS in all patients is shown in Fig. 1. The median survival time (MST) for OS was 53.3 months, and the estimated 1-, 2-, 3- and 5-year survival rates were 80.4, 61.6, 52.6, and 48.0%, respectively. DFS in all patients is shown in Fig. 2. The MST for DFS was 23.8 months, and the estimated 1-, 2-, 3- and 5-year survival rates were 64.5, 52.7, 49.2 and 47.1%, respectively.

**Figure 3:**
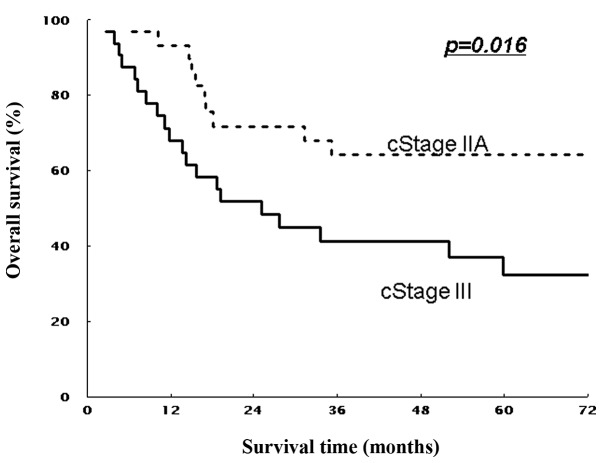
Overall survival in stage II and III patients who received preoperative chemoradiation.

**Figure 4:**
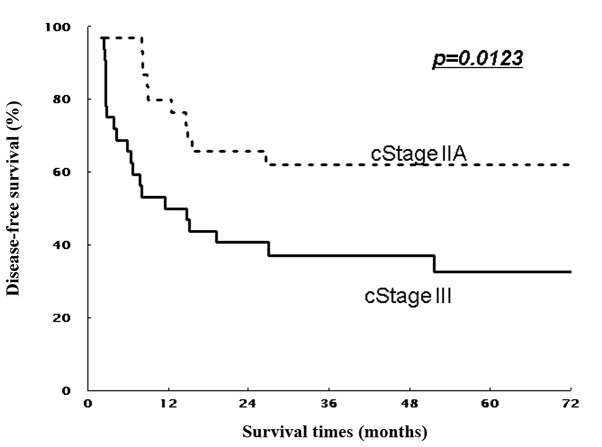
Disease-free survival in stage II and III patients who received preoperative chemoradiation.

**Figure 5:**
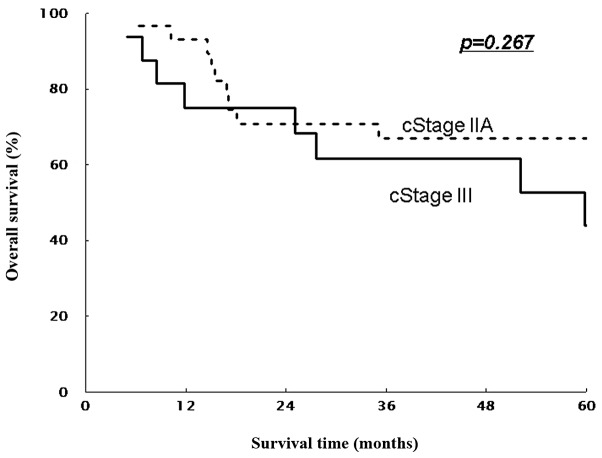
Overall survival in stage II and III patients excluding T4 patients who received preoperative chemoradiation.

**Figure 6:**
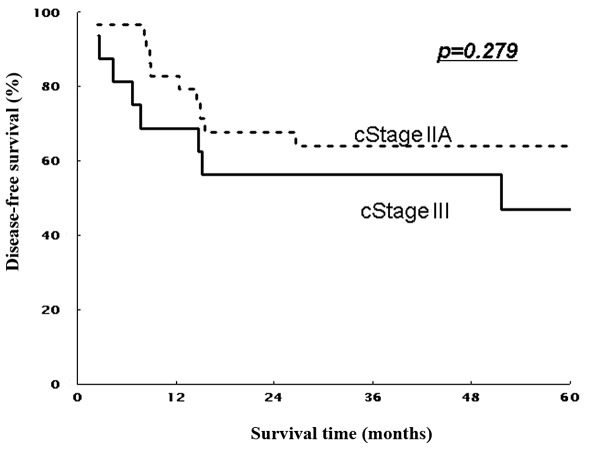
Disease-free survival in stage II and III patients excluding T4 patients who received preoperative chemoradiation.

Comparison of survival between T3 and T4 patients was additionally performed. The estimated 5-year OS rates were 63.3% for T3 patients and 28.3% for T4 patients. Similarly, the 5-year DFS rates were 61.1% for T3 patients and 26.1% for T4 patients. The clinical T3 patients showed significantly longer OS and DFS compared with clinical T4 (P=0.006 in OS and P=0.002 in DFS, respectively). Furthermore, the survival rates between different stages were compared according to the UICC Classification. The estimated 5-year OS rates were 64.2% for stage II and 33.1% for stage III (all T), and 46.9% for stage III (non-T4) patients (P=0.016 and P=0.267, Figs. 3 and 5). Similarly, 5-year DFS rates were 61.9% for stage II, 32.3% for stage III (all T), and 43.8% for stage III (non-T4) patients (P=0.011 and P=0.297; Figs. 4 and 6). Patients with stage II showed significantly longer OS and DFS than those with stage III. In subgroup analysis for stage III patients, the estimated 5-year OS and DFS were 46.9 and 43.9% for T3, and 20.3 and 18.8% for T4, respectively (P=0.045 and P=0.035, Figs. 5 and 6).

Univariate analysis for overall survival in stage II/III esophageal cancer patients is shown in Table IV. Lymph node metastasis, depth of tumor invasion and resectability showed significant differences in the prognostic value (P<0.01). Furthermore, the patients who were CRT responders showed significantly longer OS compared to those who were not (P<0.001). Using multivariate analysis, resectability and the effect of CRT were independent prognostic factors for OS (Table V).

## Discussion

We have previously reported that preoperative CRT contributes to improve the resectability in patients with ESCC, and that surgical esophagectomy remains the standard therapy for CRT responders (24). In this study, we focused on the characteristics of the UICC stage II/III ESCC, and analyzed whether this trimodality therapy combined with neoadjuvant CRT and esophagectomy improved the outcome of the patients.

This retrospective study showed that the 5-year OS rates of cstage II/III esophageal cancer patients were 64.2 and 33.1%, respectively. On the other hand, those of UICC clinical stage II/III patients after esophagectomy were reported to be from 47.5% (stage IIA) to 33.3% (stage III) by the Comprehensive Registry of Esophageal Cancer in Japan (9). These data suggest that the addition of neoadjuvant CRT is beneficial regarding the outcome of stage II patients. We failed to show the survival benefit of neoadjuvant CRT in stage III patients. However, this is thought to be due to the biased demographics in our study; more advanced T4 patients comprised approximately 50% in stage III patients. Actually, subgroup analysis showed that trimodality therapy improved the outcome of T3 more than T4 patients in stage III.

There have been nine randomized trials of preoperative CRT following surgery vs. surgery alone (15–23). Of the nine randomized trials, three studies showed survival benefits in preoperative CRT group compared to those receiving surgery alone (15,22,23). However, patient numbers in two randomized trials were too small to be evaluated objectively (15,23). Notably, a large-scale study by Burmeister *et al* revealed that 5-FU/CDDP (FP) plus radiation (35 Gy) followed by esophagectomy for ESCC improves DFS, but not for all patients including those with adenocarcinoma (22). This report has encouraged us to continue trimodality therapy for ESCC in Japan. In any case, it is difficult to evaluate these randomized studies unitarily, because all these randomized phase III reports have flaws due to their wide variation in CRT protocols, short follow-up duration, different histological types, different stages, and different operative procedures. Moreover, we are urged to standardize the regimen of chemotherapeutic agents and radiation dose. Courrech Staal *et al* systematically reviewed the benefits and risks of neoadjuvant CRT for esophageal cancer, and reported that FP was the widely used mainstay in CRT regimens all over the world (27). Therefore, it sounds reasonable that the standard chemotherapeutic regimen needs to be established based on FP regimen in Asia as well as in Western countries. The standard regimen of definitive CRT advocated by Intergroup INT0123 (RTOG9405) consists of 2 cycles of 5-FU (1,000 mg/m^2^/24 h for 4 days) and CDDP (100 mg/m^2^/bolus on Day 1) with 50.4 Gy irradiation (13). In Japan, the regimen of neoadjuvant CRT should also be determined on the basis of the INT123 study, and the concurrent radiation dose should be discussed considering the safety of surgery. In this study, CRT consists of 5-FU (500 mg/m^2^/24 h for 5 days) and CDDP (15–20 mg/bolus for 5 days) with 40 Gy irradiation as a result of discussion with radiologists. The chemotherapeutic and radiation doses in our regimen were lower than those in the INT0123 study, but our setting dose was sufficient to show the efficacy and safety with tolerability. Hospital mortality after esophagectomy following CRT was reported to be 5.2% in Courrech Staal’s review, which was compatible with that in our study (27).

The clinical response rates were assessed in this study. Those of the primary tumor ranged from 59 to 87% in previous preoperative randomized or non-randomized studies (17,18,21,28,29). Meanwhile, our study showed that the clinical response rate using the Japanese Guidelines for Esophageal Disease was 83.9% for the primary tumor and 70% for metastatic nodes.

Regarding the radiation field, the optimal radiation field design remains controversial (30–34). Hsu *et al*(30) compared the patients with AJCC stage II/III ESCC undergoing preoperative CRT (median, 36 Gy) followed by radical esophagectomy with or without elective nodal irradiation (ENI). As a result, ENI reduced the M1a failure rate, but was not associated with improved outcomes in the patients undergoing preoperative CRT. Zhao *et al*(33) also evaluated 3D-CRT (irradiating only the primary tumor and positive lymph nodes) for ESCC, and concluded that the omission of elective nodal irradiation was not associated with a significant failure in lymph node regions not included in the planned target volume. In our study, we planned a radiation field including both the primary tumor and metastatic lymph nodes which were identified by an enhanced CT scan. Namely, we planned the irradiation field minimally to prevent operative and postoperative complications. Consequently, CRT minimized postoperative complications as we expected and improved the prognosis beyond our expectations, especially with the marked clinical response for metastatic nodes. In China, Zhao *et al* also used the same radiation field setting (33). In this way, the minimum setting for the primary tumor and metastatic nodes may be promising to achieve fewer complications and more prognostic benefits.

A recent meta-analysis revealed that a significant survival benefit for neoadjuvant CRT was evident for patients with resectable esophageal cancer with no increase in the morbidity rate [hazard ratio (HR), 0.81], and that definitive CRT did not demonstrate any survival benefit over other curative strategies (35). Intriguingly, neoadjuvant chemotherapy (without radiation) did not show any survival benefit (HR, 0.93). In Japan, preoperative chemotherapy with FP has been regarded as the standard treatment for patients with stage II/III (non-T4) ESCC by the JCOG 9204 and 9907 trials (36,37). However, some critical problems were pointed out in these prospective randomized studies. First, there was a significant difference in subject numbers between pre- and postoperative chemotherapy groups (P=0.04) Secondly, patients with the pN0 status did not undergo postoperative chemotherapy in reality. Therefore, future clinical trials should resolve these above-mentioned problems. The 5-year OS in stage II/III (T3) patients in our study was higher than that in the JCOG study (63.3 vs. 55%, respectively). We strongly propose that preoperative CRT be included in the next JCOG study to evaluate the efficacy of CRT more objectively in Japan.

In conclusion, preoperative CRT for cstage II/III (non-T4) ESCC patients contributed to high response rates for both the primary tumor and metastatic nodes and showed satisfactory outcome with tolerable morbidity and mortality. A phase II study is needed to better clarify the standard neoadjuvant CRT regimen through a large prospective randomized trial.

## Figures and Tables

**Table I:** Patient characteristics.

	Stage II (n=30)	Stage III (n=32)
Age, mean	60.33	61.32
Male/Female	26/4	24/8
Location of primary tumor		
Cervical		2
Upper thoracic	3	4
Middle thoracic	22	17
Lower thoracic	5	7
Abdominal		2
T-classification		
T3	30	15
T4		17
N-classification		
N0	27	16
N1	3	16

**Table II:** Effects of CRT for primary tumor and metastatic nodes.

Response	Primary tumor	Metastatic nodes	Clinical response rate (Primary tumor and metastatic nodes)
CR, n	14	4	14
PR, n	38	10	37
NC, n	9	4	9
PD, n	1	2	2
Response rate (%)	83.9	70	82.3

CR, complete response; PR, partial response; NC, no change; PD, progressive disease.

**Table III:** Postoperative complications after esophagectomy for patients with stage II, III esophageal cancer.

Complications	n (%)
Anastomotic leakage	6 (9.7)
Recurrent nerve palsy	4 (6.5)
Respiratory failure	4 (6.5)
Pleural effusion	2 (3.2)
Sepsis	1 (1.6)
Arrhythmia	1 (1.6)
Myocardial infarction	1 (1.6)
SMA occlusion	1 (1.6)

SMA, superior mesenteric artery.

**Table IV:** Univariate analysis for OS.

Characteristics	No. of patients	Hazard ratio	OS P-value	95% CI
Age (years)				
<70	48	1.367	0.53	0.514–3.65
≥70	14			
Gender				
Male	51	0.841	0.489	0.515–1.373
Female	11			
Effect of CRT				
Effective	51	0.29	0.00051^b^	0.091–0.476
Not effective	11			
Lymph nodes metastasis				
Positive	19	2.855	0.00075^b^	1.3–6.27
Negative	43			
Depth of tumor invasion				
T3	45	3.463	0.0078^b^	1.55–7.719
T4	17			
Tumor location^a^				
Upper	9	0.767	0.628	0.262–2.241
Lower	53			
Counts of lymph nodes metastasis				
>4	6	23.77	0.00001^b^	6.93–81.57
<3	56			
Resectability				
R0	45	10.23	0.00001^b^	4.34–24.1
R1, R2	17			
Pathological complete response				
Yes	15	25.17	0.005^b^	6.83–43.5
No	47			

a Upper, tumor located above the bifurcation; lower, below the bifurcation.

b Statistically significant.

**Table V:** Multivariate analysis of factors associated with OS of ESCC.

	P-value	HR	95% CI
Age	0.28	1.633	0.671–3.972
Lymph nodes metastasis	0.659	0.819	0.337–1.990
Depth of tumor invasion	0.126	2.155	0.805–5.770
Clinical stage	0.752	1.176	0.432–3.199
Resectability	0.001^a^	5.072	2.059–12.497
Effect of CRT	0.01^a^	0.279	0.106–0.733

a Statistically significant.

HR, hazard ratio; CI, confidence interval.
